# The Impact of Metformin on Vitamin B12 Levels in Children and Adolescents: A Systematic Review and Single‐Arm Meta‐Analysis

**DOI:** 10.1002/edm2.70232

**Published:** 2026-05-17

**Authors:** Einas Abdullah Tahir, Muhtadi G. Ahmed, Motasam Belah Al Swayah, Sarah Mostafa Elsayed, Farhan Ahmed Alnoaimi, Ahmed Mohamed Hegazy, Nada Sayed Abdelalim, Rehab Adel Diab

**Affiliations:** ^1^ Faculty of Medicine University of Khartoum Khartoum Sudan; ^2^ Faculty of Medicine Mansoura University Mansoura Egypt; ^3^ Faculty of Medicine University of Tripoli Tripoli Libya; ^4^ Faculty of Medicine Al‐Azhar University Cairo Egypt; ^5^ Faculty of Medicine Cairo University Cairo Egypt; ^6^ Acute Medicine Medway NHS Foundation Trust Gillingham UK; ^7^ Faculty of Medicine Fayoum University Fayoum Egypt; ^8^ Cardiology Department, Faculty of Medicine Al‐Azhar University Cairo Egypt

## Abstract

**Introduction:**

The use of metformin among children and adolescents has risen in the past decade; however, its effect on vitamin B12 has been unclear. To address this gap, we conducted this first systematic review and single‐arm meta‐analysis.

**Objective:**

To investigate the effect of metformin on vitamin B12 levels.

**Methods:**

PubMed, Scopus and Web of Science were systematically searched until April 2025. The primary outcomes were the effect of metformin on vitamin B12 levels after 6 and 12 months of duration, and vitamin B12 deficiency after at least 12 months of usage. We conducted the risk‐of‐bias assessment using the NOS and Cochrane ROB2 tool. The mean difference (MD) and standard error (SE) were calculated and used for analysis. We assessed the heterogeneity of the studies using the chi‐squared test and measured it with the *I* ^2^ statistic. A fixed effects model was applied to calculate the pooled MD in the absence of significant heterogeneity. The registration number in PROSPERO 2025 is CRD420251045023.

**Results:**

A total of five articles were included in this systematic review and meta‐analysis. The pooled rate of vitamin B12 deficiency in RCT studies after at least 12 months of use was 1.4% [95% CI: −0.012, 0.040] with mild heterogeneity (*I* ^2^ = 30.87%, *p* = 0.235). While in observational studies the pooled rate was 18.7% [95% CI: −0.212, 0.586] with substantial heterogeneity (*I* ^2^ = 87.93%, *p* = 0.004). Vitamin B12 levels decreased after 6 and 12 months of using metformin; however, it was neither statistically nor clinically significant (*p* > 0.05).

**Conclusion:**

More trials with larger sample sizes and longer durations of follow‐up are needed to better understand the risk of vitamin B12 deficiency among the paediatric population.

**Trial Registration:** PROSPERO (CRD420251045023).

## Graphical abstract

This first systematic review and single‐arm meta‐analysis in children and adolescents taking metformin found a significant vitamin B12 deficiency rate in observational studies than in randomised controlled trials (RCT). Larger RCTs with longer duration are recommended to better understand the risk.

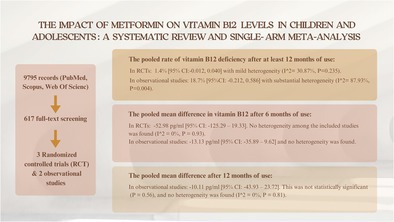

## 1 Introduction

Metformin was first investigated in the 1950s by Jean Sterne as a potential replacement for insulin in some patients with maturity‐onset and juvenile‐onset diabetes. In the past century, metformin has progressed from a newly discovered drug to the first‐line oral hypoglycaemic agent for the management of type 2 diabetes mellitus (T2DM) [1]. Further research was conducted to understand the mechanism of action of metformin, which revealed that it decreases hepatic glucose synthesis and intestinal glucose absorption as well as increasing insulin sensitivity. Some classes have the ability to lower blood glucose with risk of causing hypoglycaemia by increasing insulin secretion. However, it is distinct from other classes of oral antidiabetes agents such as sulphonylureas [2]. In addition to diabetes, metformin has extra‐glycaemic clinical benefits, including endothelial protection [3], antineoplasm [4] and anti‐ageing/anti‐inflammation [5]. It may also assist in ovulatory abnormalities in girls with polycystic ovary syndrome (PCOS) [6]. Metformin is generally considered safe and well‐tolerated. However, a large proportion of patients cannot tolerate it due to its associated side effects, such as diarrhoea, nausea and vomiting, which are relatively common and affect up to 30% of patients taking metformin [7]. In addition, the most serious complication of metformin is metformin‐associated lactic acidosis (MALA) [8]. MALA is a rare side effect that occurs during hypoxia, renal impairment and hypovolaemia including severe dehydration and shock. Over the last few decades, several studies have reported a relationship between metformin use and vitamin B12 deficiency [9]. Vitamin B12 is a cofactor for enzymes that play a role in DNA synthesis and neuronal protection. Therefore, vitamin B12 deficiency can lead to haematological and neurological disorders such as megaloblastic anaemia and neuropathy. However, among the paediatric population, the effect of metformin on vitamin B12 levels remains unclear. Hence, it is important to understand this relationship. This study aims to investigate the effect of metformin on serum vitamin B12 levels among the paediatric population, particularly with respect to treatment duration. Understanding this relationship would help clinicians anticipate and monitor the side effects of metformin and optimise its dosage for safer and more effective use in children.

## 2 Methods

### 2.1 Study Selection

This meta‐analysis was done according to the Preferred Reporting Items for Systematic Reviews and Meta‐Analyses statement (PRISMA) guidelines and we followed the Cochrane handbook of systematic reviews and meta‐analysis of interventions (version 5.1.0). The protocol is registered in the international systematic review registry PROSPERO under CRD420251045023.

### 2.2 Eligibility Criteria

We included studies that met all of the following criteria: (1) *Population*: Studies involving children and adolescents (< 18 years). (2) *Intervention*: Studies reporting the use of metformin. (3) *Outcome*: Studies presenting serum vitamin B12 levels as an outcome.

In addition, we included randomised controlled trials (RCTs), non‐RCTs, observational cohort studies and case–control studies and studies published in peer‐reviewed journals indexed in major databases. We excluded studies that met the following criteria: (1) studies involving adult patients on metformin; (2) reviews, meta‐analyses, case reports, case series, cross‐sectional studies, editorials, letters and conference abstracts. The registered protocol initially planned to exclude studies where participants taking vitamin B12 supplements were excluded. However, due to the limited eligible studies in paediatrics on this topic, and to avoid loss of relevant data, this criteria was modified.

### 2.3 Literature Search

We searched PubMed, Scopus and Web of Science (WOS), from inception until April 2025, and we used the following search strategy ((Children OR Adolescents OR pediatric* OR Paediatric) AND ((Metformin) OR (Glucophage) OR (Biguanides))).

### 2.4 Selection Process and Data Extraction

We removed the duplicates using EndNote software, and at least two reviewers screened the title and abstract using Rayyan software, and later it was followed by full‐text screening of the included studies by at least two independent authors. Disagreements at any stage were resolved through discussion with a third reviewer. All reviewers agreed to the extraction of data.

### 2.5 Data Extraction

Two reviewers independently extracted the data using a predesigned standardised Excel spreadsheet. The data included study features (e.g., publication year, study design, country or region and metformin doses, etc.), baseline patient characteristics (e.g., BMI, gender, glucose levels) and outcome data. Information for assessing the risk of bias was also extracted. Any disagreements were addressed through discussions with a third reviewer to achieve consensus.

### 2.6 Data Analysis

The study outcomes include the change in vitamin B12 levels over 6 and 12 months. A single‐arm meta‐analysis was done to estimate the pooled mean difference (MD) across the studies. In each study, the MD and standard error (SE) were calculated and used for analysis.

We assessed the heterogeneity of the studies using the chi‐squared test and measured it with the *I* ^2^ statistic. A fixed effects model was applied to calculate the pooled MD in the absence of significant heterogeneity; if heterogeneity was found, a random effect model was used. All statistical analyses were done using Review Manager (RevMan) version 5.4.1. and Open meta‐analyst, and a *p*‐value below 0.05 was considered statistically significant.

### 2.7 Quality Assessment

Two independent reviewers evaluated the risk of bias for all included studies. Any disagreements were resolved through discussion or, when required, by a third reviewer. Randomised clinical trials were assessed using the Cochrane risk‐of‐bias tool (ROB2) [10]. The ROB2 assessed five domains: randomisation process, deviations from intended interventions, missing outcome data, outcome measurement and selection of reported results. Each domain was judged as low, moderate or high risk. Studies were considered to be of high risk if one of the domains was high. For observational studies, the Newcastle–Ottawa Scale (NOS) was applied [11], covering three sections: selection, comparability and outcome assessment. The scale total point was 9: 4 points for selection, 2 for comparability and 3 for outcomes. Studies achieving 7–9 points were categorised as high quality, 4–6 as moderate quality and 0–3 as low quality.

## 3 Results

### 3.1 Study Selection

**FIGURE 1:**
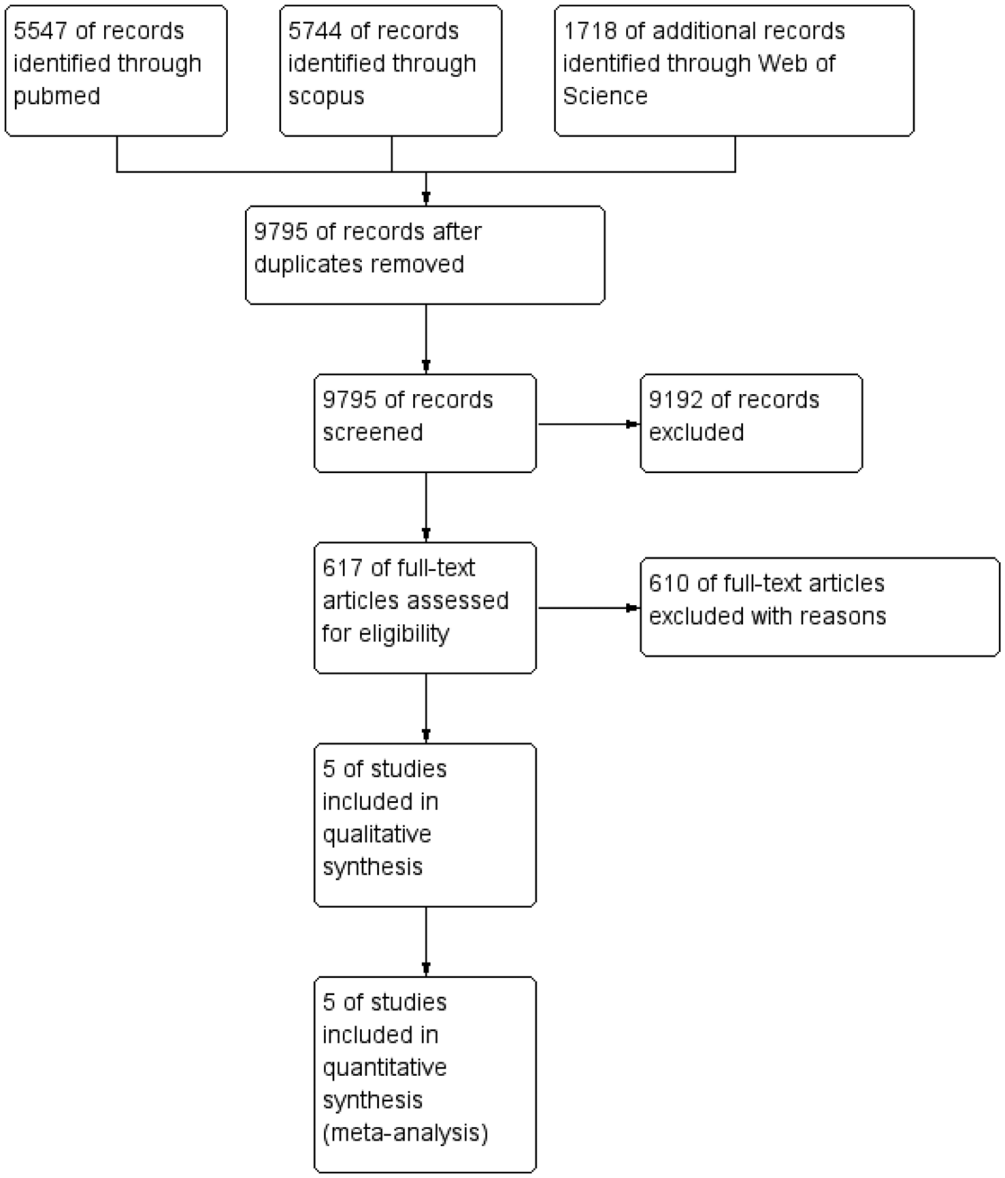
Prisma flow diagram of the study selection for this systematic review and meta‐analysis.

The initial database search yielded 13,009 results, of which 3214 duplicates were removed. We reviewed the titles and abstracts of the remaining 9795 studies, which reduced the number to 617. We then conducted a full‐text screening of these studies and excluded 611 of them. As a result, only five studies were deemed eligible and included in the meta‐analysis [12, 13, 14, 15, 16] (Figure 1).

### 3.2 Study Characteristics

We included five studies in this systematic review and meta‐analysis, as summarised in Tables 1 and 2. Three were RCTs and two were single‐arm observational studies [9, 10, 11, 12, 13, 14]. The studies involved paediatric populations aged 6–18 years with conditions such as type 2 diabetes, obesity, insulin resistance and metabolic syndrome. Across all studies, metformin was administered at doses ranging from 500 to 2000 mg daily. We identified a total of 307 patients who received metformin treatment. Follow‐up durations ranged from 6 months to 3 years. In Taş et al. [13] patients included in the study did not receive any vitamin supplements during the study. In addition, Yu et al. [16], Anderson et al. [12] and van der Aa et al. [14] mentioned that children who were taking vitamin B12 or multivitamin supplements were excluded; however, it was not clear if they received any supplements during the study. Nevertheless, patients participating in Yanovski et al. [15] received daily cyanocobalamin‐containing multivitamin (6 mg cyanocobalamin), and despite this, patients taking metformin in high doses (2000 mg) showed vitamin B12 deficiency. The baseline B12 levels ranged from 211.38 to 683 pg/mL. We reported the levels of vitamin B12 levels after 6 months of use, where all studies showed reduction ranging from −1.35 to −57.1 pg/mL. In addition, after 12 months of use, the reduction ranged from −4.92 to −90.79 pg/mL. We also reported vitamin B12 deficiency after at least 12 months of use, where 9 participants out of 284 (3.1%) in the metformin group have shown deficiency in vitamin B12 (Table S1).

**TABLE 1:** Summary of the characteristics and outcomes of the included studies in this systematic review and meta‐analysis.

Study ID	Title	Study design	Country	Sample size (*N*)	Follow‐up period	Metformin dose	Diagnosis of vitamin B12 deficiency	Inclusion criteria	Exclusion criteria	Primary outcome	Secondary and safety outcomes	Vitamin B12 results
Anderson et al. [12]	Effect of metformin on vascular function in children with type 1 diabetes	Randomised double‐blind placebo‐controlled trial	Australia	90	12 months	500 mg twice a day or 1000 mg twice a day, depending on body weight		Patients diagnosed with type 1 diabetes at least 6 months prior, required > 0.5 units of insulin/kg/day, aged 8–18 years and for age and sex, BMI greater than 50th centile.	Patients who had a severe hypoglycaemic event in 6 months prior to recruitment, more than 2 events of diabetic ketoacidosis in the previous 12 months, serious comorbidities, have contraindications to metformin therapy, or were already using metformin, statins, multivitamins or antihypertensive drugs	Flow‐mediated dilatation (FMD)	GTN‐mediated dilation, HbA1c, insulin dose, estimated insulin sensitivity, leptin, adiponectin, BMI, waist circumference, hip circumference, waist–hip ratio, systolic and diastolic blood pressure, lipid profile, carotid and aortic IMT, fat mass, side effects, vitamin B12	Vitamin B12 levels were lower in the metformin group but remained within the normal reference range
Taş et al. [13]	Does metformin treatment in paediatric population cause vitamin B12 deficiency?	Single‐arm prospective observational study	Turkey	24	6 and 12 months	1000 mg/day	B12 < 150 pmol/L	Patients with metabolic syndrome, type 2 diabetes mellitus, polycystic ovarian syndrome	Patients with any disease that could affect vitamin B12 levels or could cause vitamin B12 deficiency, vegetarians and those who received H2‐receptor antagonists, proton pump inhibitors or vitamin supplements in the past 6 months	Vitamin B12, homocysteine, methylmalonic acid (MMA), holo‐transcobalamin II (holo‐TC‐II), haemoglobin, MCV, RDW	Hb, MCV, RDW	Metformin did not significantly affect vitamin B12 status over 12 months in paediatric patients
van der Aa et al. [14]	Long‐term treatment with metformin in obese, insulin‐resistant adolescents	Randomised double‐blind placebo‐controlled trial	the Netherlands	62 (42 completed)	18 months	2000 mg/day	B12 < 140 pmol/L	Age 10–16 years; BMI‐SDS > 23, HOMA‐IR 23.4; Caucasian descent; written informed consent	T2DM: PCOS: Endocrine disorders treated with corticosteroids; heights 1.3 SD from target height; syndromal disorders, pregnancy: History of alcohol abuse, impaired renal function (GFR < 80mlmlo); impaired hepatic function (ALT > 150% of normal value for age) insufficient knowledge of Dutch language	BMI, HOMA‐IR	Fat mass, fat‐free mass, body fat %, HbA1c, renal and hepatic function, gastrointestinal side effects, quality of life, physical fitness, vitamin B12	Some participants had decreased vitamin B12 levels
Yanovski et al. [15]	Effects of metformin on body weight and body composition in obese insulin‐resistant children	Randomised double‐blind placebo‐controlled trial	USA	100	6 months + 6‐month open‐label	1000 mg twice per day	B12 < 132.8 pmol/L	Obese children, aged 6–12 years, were eligible if they had BMI $95th percentile were prepubertal or early pubertal and had fasting hyperinsulinaemia	Impaired fasting glucose, were diabetic, or reported a diagnosed renal, cardiac, endocrine, pulmonary, or hepatic disease that might alter body weight. Subjects were excluded for baseline creatinine 0.1 mg/dL and for alanine aminotransferase (ALT) or aspartate aminotransferase (AST) that exceeded 1.5 times the upper limit of the laboratory normal range	BMI *z*‐score	MI, body weight, fat mass, insulin resistance (HOMA‐IR), fasting glucose, metabolic syndrome, adverse events, GI symptoms, vitamin B12	Serum vitamin B12 remained within normal range but declined in the metformin group compared with placebo
Yu et al. [16]	Effect of metformin on vitamin B12 levels in paediatric patients	Retrospective observational single arm cohort study	USA	151	36 months	Ranged from 500 to 2000 mg/day		Patients aged 6–17 years	If prior to treatment children were hypercobalaminaemic (> 950 pg/mL) or hypocobalaminaemic (< 200 pg/mL) or if they were taking vitamin B12 or multivitamin supplements	Vitamin B12 level	Effect of dose and compliance on vitamin B12	Vitamin B12 levels generally remained within normal range. Only high‐dose + good compliance led to significant decreases at 24 and 36 months, but deficiency was rare

*Note:* Vitamin B12 deficiency was defined according to the diagnostic cut‐off reported in each study.

Abbreviations: ALT = alanine aminotransferase; AST = aspartate aminotransferase; BMI = body mass index; BMI‐SDS = body mass index standard deviation score; GFR = glomerular filtration rate; GTN = glyceryl trinitrate‐mediated dilatation; Hb = haemoglobin; HbA1c = glycosylated haemoglobin; HOMA‐IR = homeostatic model assessment of insulin resistance; IMT = intima‐media thickness; MCV = mean corpuscular volume; PCOS = polycystic ovarian syndrome; RCT = randomised controlled trials; RDW = red cell distribution width; SD = standard deviation; T2DM = type 2 diabetes mellitus.

**TABLE 2:** Baseline characteristics of the included studies in this systematic review and single‐arm meta‐analysis.

Study ID		Sample size	Male, *N* (%)	Glucose (mmol/L)	Baseline vitamin B12 (pg/mL) mean ± SD
Anderson et al. [12]	Metformin	45	87.50%	10 ± 4	506.98 ± 227.64
	Placebo	45	80%	12 ± 5	646.34 ± 245.26
Taş et al. (6 months treated group) [13]	Metformin	24	37.5%	—	211.38 ± 59.62
				—	
Taş et al. (12 months treated group) [13]	Metformin	11		—	222.22 ± 50.14
				—	
van der Aa et al. [14]	Metformin	23	26.10%	4.8	475.16 ± 163.81
	Placebo	19	42.10%	4.8	489.16 ± 85.23
Yanovski et al. [15]	Metformin	53	43%	5.1 ± 0.4	683 ± 270
	Placebo	47	36%	5.1 ± 0.4	694 ± 265
Yu et al. [16]	Metformin	151	21.2%	—	—
	Placebo		—	—	—

Abbreviations: SD = standard deviation, mmol/L= millimoles per liter.

## 4 Risk‐of‐Bias Assessment

Results of the risk‐of‐bias assessment are summarised in Tables 4. The risk of bias in the RCTs were evaluated using the Cochrane ROB2 assessment tool, as presented in Table 3. Anderson et al. [12], Van der et al. [14] and Yanovski et al. [15] were judged as low risk. The observational studies were assessed using NOS, as shown in Table 4. The total NOS score for Taş et al. [13] was 6, indicating moderate methodological quality, whereas the score for Yu et al. [16] was 8, indicating high quality.

**TABLE 3:** Risk‐of‐bias assessment of the randomised controlled trials using ROB2 assessment tool.

Study ID	ROB2 sequence generation	ROB2 allocation concealment	ROB2 blinding of the study patients	ROB2 blinding of outcome	ROB2 incomplete outcome data	ROB2 selective outcome reports	Overall risk
Anderson et al. [12]	Low	Low	Low	Low	Low	Low	Low
Van der et al. [14]	Low	Low	Low	Low	Low	Low	Low
Yanovski et al. [15]	Low	Low	Low	Low	Low	Low	Low

Abbreviation: ROB2 = risk‐of‐bias tool.

**TABLE 4:** Risk‐of‐bias assessment of the observational studies using the Newcastle–Ottawa quality assessment tool.

Study ID	Representativeness of the exposed cohort	Selection of the non‐exposed cohort	Ascertainment of exposure	Outcome of interest was not present at start of study	The design or analysis is controlled for confounders	Assessment of outcome	Follow‐up was long enough for outcomes to occur	Follow‐up of cohort was adequate	Overall score
Taş et al. [13]	*			*	**	*	*		6
Yu et al. [16]	*		*	*	**	*	*	*	8

*Note:* * Indicates one point. ** Indicates two points.

## 5 Meta‐Analysis

### 5.1 Meta‐Analysis of Vitamin B12 Deficiency After at Least 12 Months of Use

**FIGURE 2:**
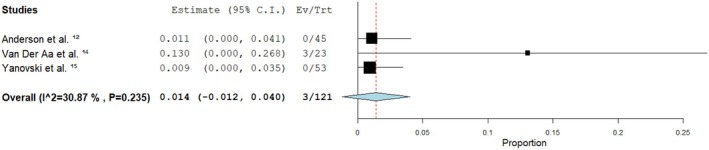
Forest plot showing pooled effect of metformin on vitamin B12 levels after at least 12 months of use in randomised clinical trials. *I* ^2^ indicates heterogeneity across studies.

**FIGURE 3:**
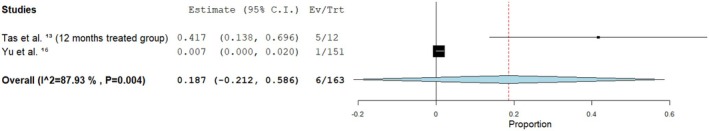
Forest plot showing the pooled effect of metformin on vitamin B12 levels after at least 12 months of use in observational studies. *I* ^2^ indicates heterogeneity across studies.

Due to the differences in the study designs we classified the analysis into RCTs and observational studies. Among the randomised clinical trials (RCT) the pooled rate of vitamin B12 deficiency after at least 12 months was 1.4% [95% confidence interval (95% CI): −0.012, 0.040] with mild heterogeneity (*I* ^2^ = 30.87%, *p* = 0.235), however it was not statistically significant (Figure 2). While in the observational studies the pooled rate was 18.7% [95% CI: −0.212, 0.586] with substantial heterogeneity (*I* ^2^ = 87.93%, *p* = 0.004) and it was statistically significant (Figure 3).

**FIGURE 4:**
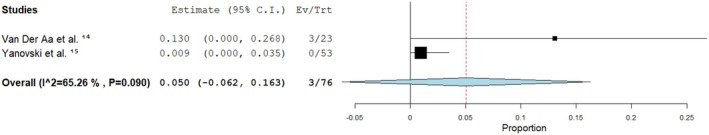
Forest plot showing the pooled effect of vitamin B12 deficiency after at least 12 months of using high doses of metformin in RCT. *I* ^2^ indicates heterogeneity across studies.

We also performed a subgroup analysis in RCTs to understand the relationship between high‐dose of metformin (more than 1000 mg/day) on longer duration of use (i.e., more than or equal to 18 months of use). The analysis showed substantial heterogeneity (*I* ^2^ = 65.26%, *p* = 0.090) and the pooled deficiency rate was 5% [95% CI: −0.062, 0.163]. However, it was not statistically significant (Figure 4).

#### 5.1.1 Sensitivity Analysis

We conducted a sensitivity analysis using leave‐one‐out analysis for the meta‐analysis of serum vitamin B12 levels after at least 12 months of use, where studies were removed one at a time and the pooled estimate was recalculated. The pooled estimates ranged from 0.009 to 0.037 and all 95% CIs included the null, hence it was not statistically significant. Exclusion of Yu et al. [16] study yielded a pooled estimate of 0.037 (95% CI −0.016, 0.090), with substantial heterogeneity (*I* ^2^ = 72.55%, *p* = 0.012) (Figure S1). Similarly, exclusion of Yanovski et al. [15] resulted in a pooled estimate of 0.030 (95% CI −0.016, 0.075), with also substantial heterogeneity (*I* ^2^ = 73.55%, *p* = 0.010) (Figure S2). However, when excluding Taş et al. [13] the pooled estimate was 0.009 (95% CI −0.003, 0.020) with reduced heterogeneity (*I* ^2^ = 3.94%, *p* = 0.373) (Figure S3) In addition, exclusion of van der Aa et al. [14] and Anderson et al. [12] resulted in a pooled estimate of 0.012 (95% CI −0.013, 0.036) and 0.025 (95% CI −0.015, 0.066), respectively, with persistent heterogeneity (Figures S4 and S5).

### 5.2 Meta‐Analysis of the Change in B12 After 6 Months

**FIGURE 5:**
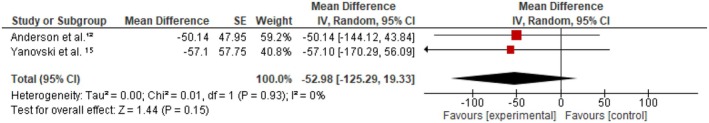
Forest plot showing the pooled effect estimate of randomised controlled trials after 6 months of metformin use. *I* ^2^ indicates heterogeneity across studies.

**FIGURE 6:**
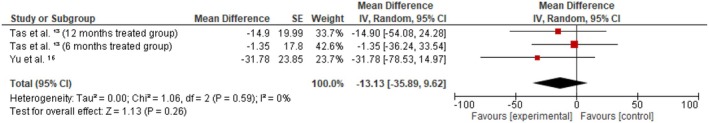
Forest plot showing the pooled effect estimate of observational studies after 6 months of metformin use. *I* ^2^ indicates heterogeneity across studies.

Four studies were included in this analysis. We have also classified the analysis by study design into RCTs and observational studies. For RCTs, the pooled MD was −52.98 pg/mL [95% CI: −125.29, 19.33]. No heterogeneity among the included studies was found (*I* ^2^ = 0%, *p* = 0.93) (Figure 5). For observational studies, the pooled MD was −13.13 pg/mL [95% CI: −35.89, 9.62] and no heterogeneity was found (*I* ^2^ = 0%, *p* = 0.59) (Figure 6). While both groups showed a reduction in vitamin B12 levels, the results were not statistically significant in either subgroup.

### 5.3 Meta‐Analysis of the Change in Vitamin B12 After 12 Months

**FIGURE 7:**
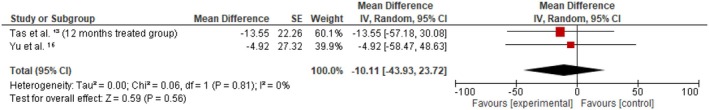
Forest plot showing the pooled effect estimate of observational studies after 12 months of metformin use. *I* ^2^ indicates heterogeneity across studies.

The analysis of change in vitamin B12 levels after 12 months was also classified by study design. Two observational studies were pooled, reporting a MD of −10.11 pg/mL [95% CI: −43.93, 23.72]. The test for overall effect suggested that this observed change was not statistically significant (*p* = 0.56), and no heterogeneity was found (*I* ^2^ = 0%, *p* = 0.81) (Figure 7). For RCTs, data at 12 months were limited to one study [12], which reported a MD of −90.79 pg/mL [95% CI: −202.94, 21.36]. This reduction was also not statistically significant.

## 6 Discussion

### 6.1 Significance and Finding of the Study

This systematic review and meta‐analysis is the first to assess the effect of metformin on vitamin B12 levels in the paediatric population. The primary outcome was the effect of metformin on vitamin B12 levels after 6 and 12 months duration, and vitamin B12 deficiency after at least 12 months of use. A meta‐analysis of five studies after at least 12 months of metformin use showed a pooled prevalence of vitamin B12 deficiency of 1.4% in RCT with mild heterogeneity, while in observational studies, the pooled prevalence was 18.7% with substantial heterogeneity. All studies reported a decline in vitamin B12 levels after 6 and 12 months of use, however this decline was within the reference range, hence, neither clinically nor statistically significant in the meta‐analysis (i.e., *p* > 0.05).

### 6.2 Agreement and Disagreement With Previous Study

While several randomised clinical studies have been conducted to study this effect among adults [17], data have been limited in the paediatric population. The pooled prevalence of vitamin B12 deficiency in children and adolescents was lower in RCT (i.e., 1.4%) than the findings in the meta‐analysis done in the adult population [17], where 10.7% of the participants in the metformin group of the later study had vitamin B12 deficiency. However, in observational studies the pooled prevalence was higher (i.e., 18.7%) with substantial heterogeneity but due to the limited number of studies a subgroup analysis was not conducted. This discrepancy in findings among different study designs is concerning as even a small percentage in vitamin B12 deficiency in the paediatric population can have serious neurological symptoms like developmental delay, seizures and hypotonia [18, 19], and in older children, vitamin B12 deficiency is associated with higher rates of school absenteeism and grade repetition [20]. Moreover, neurological effects in the paediatric population can cause irreversible damage due to the earlier developmental and growth stage at which the deficiency occurs [21, 22].

### 6.3 Explanation of Results

The findings of vitamin B12 levels after 6 and 12 months of use could be attributed to the short duration of treatment, where most of the included studies had an average follow‐up duration of 12 months with metformin doses ranging from 500 to 2000 mg/day. This is also supported by the findings in Yu et al. [16], where the study reported significant changes in B12 levels after 3 years of using high doses of metformin (i.e., > 1000 mg/day). Consistent with these findings, our subgroup analysis further indicated that heterogeneity was partly driven by dosage differences. High‐dose regimens (> 1000 mg/day) for at least 12 months of metformin use was associated with a 4.9% deficiency rate. While this finding highlighted a potential dose‐dependent pattern in B12 variability, it was not statistically significant. This could be due to the small sample size (*N* = 307) in comparison with the meta‐analysis in adults, where 4070 participants were included in the metformin group of the later study.

Another point that should be considered is the diagnostic cutoff point used in the paediatric population. A recent study reported neurological findings in adolescents with vitamin B12 levels considered low‐normal using the diagnostic cutoff in adults with values as high as 169 and 262 pmol/L [23, 24]. The included studies in our meta‐analysis have reported vitamin B12 deficiency when levels were less than 150 pmol/L (Table 1). These variations raise concern that deficiencies reported may have not reflected the actual magnitude of vitamin B12 deficiency, hence leading to underdiagnosis accordingly. Additionally, this highlights the importance of further research in this area to understand if modification in current cutoff values for vitamin B12 are required. Nevertheless, standardising the diagnostic vitamin B12 threshold is important to properly accommodate children and adolescents, hence preventing late diagnosis and underdiagnosis.

### 6.4 Limitations

The small number of studies eligible limited our sample size and statistical power. This is probably due to the late rise of metformin use among the paediatric and adolescent population, which only occurred recently within the last 10 years for various diseases including obesity, prediabetes, type 1 diabetes and PCOS [25, 26, 27]. Another study limitation is the differences in the metformin doses across studies. Some studies used metformin doses ranging from 500 to 1000 mg/day [12, 13], while others used higher doses ≥ 1700 mg/day [14, 15, 16]. In addition, there were irregularities in reporting the use of vitamin B12 supplements during the studies.

### 6.5 Summary

Although there are few limitations, this meta‐analysis is the first to assess the relationship between metformin and vitamin B12 levels in the paediatric population. All objectives of this systematic review and meta‐analysis have been answered. Our findings suggest deficiency in vitamin B12 levels after at least 12 months of use; hence, more randomised controlled studies with consistent doses of metformin are needed to better understand the effect and prevent under diagnosis.

## 7 Conclusion

This systematic review and meta‐analysis reports a 1.4% deficiency rate in vitamin B12 levels after at least 12 months of use in RCT and an 18.7% deficiency rate in observational studies. It also shows a decline in vitamin B12 levels after 6 and 12 months of use; however, these findings were neither statistically nor clinically significant. This is likely due to the short duration of treatment and the small sample size. More trials with larger sample sizes, consistent and standardised doses of metformin, and longer duration of follow‐up are needed to better understand the risk and magnitude of vitamin B12 deficiency among the paediatric population. In addition, a standardised vitamin B12 deficiency cutoff value should be considered to prevent under and over‐diagnosis.

## Author Contributions

**Motasam Belah Al Swayah:** methodology, formal analysis, writing – original draft, investigation, software, data curation. **Einas Abdullah Tahir:** methodology, data curation, formal analysis, writing – original draft, writing – review and editing, project administration, supervision, conceptualization, validation, investigation, software, visualization. **Muhtadi G. Ahmed:** methodology, data curation, formal analysis, software, writing – review and editing. **Farhan Ahmed Alnoaimi:** writing – original draft, writing – review and editing, data curation, software, methodology. **Rehab Adel Diab:** supervision, validation, project administration, writing – review and editing, methodology. **Sarah Mostafa Elsayed:** investigation, writing – original draft, writing – review and editing, data curation, software. **Ahmed Mohamed Hegazy:** methodology, data curation, investigation, software. **Nada Sayed Abdelalim:** writing – original draft, writing – review and editing, data curation.

## Funding

The authors have nothing to report.

## Ethics Statement

The authors have nothing to report.

## Conflicts of Interest

The authors declare no conflicts of interest.

## Supporting information


**Table S1:** Outcomes of the included studies in this systematic review and meta‐analysis.
**Figure S1:** Forest plot showing the pooled effect estimate after excluding Yu et al. [16] study in the leave‐one‐out sensitivity analysis. *I* ^2^ indicates heterogeneity across studies.
**Figure S2:** Forest plot showing the pooled effect estimate after excluding Yanovski et al. [15] study in the leave‐one‐out sensitivity analysis. *I* ^2^ indicates heterogeneity across studies.
**Figure S3:** Forest plot showing the pooled effect estimate after excluding 12 months outcome from Taş et al. [13] study in the leave‐one‐out sensitivity analysis. *I* ^2^ indicates heterogeneity across studies.
**Figure S4:** Forest plot showing the pooled effect estimate after excluding van Der Aa et al. [14] study in the leave‐one‐out sensitivity analysis. *I* ^2^ indicates heterogeneity across studies.
**Figure S5:** Forest plot showing the pooled effect estimate after excluding Anderson et al. [12] study in the leave‐one‐out sensitivity analysis. *I* ^2^ indicates heterogeneity across studies.

## Data Availability

The data that support the findings of this study are available from the corresponding author upon reasonable request.
