# Supplementary material for: The Impact of Metformin on Vitamin B12 Levels in Children and Adolescents: A Systematic Review and Single‐Arm Meta‐Analysis
Source: Endocrinol Diabetes Metab. 2026 May 17;9(3):e70232. doi: 10.1002/edm2.70232 (PMC13181207; doi:10.1002/edm2.70232)
Supplement: Supplementary file 1 — Table S1: Outcomes of the included studies in this systematic review and meta‐analysis. Figure S1: Forest plot showing the pooled effect estimate after excluding Yu et al. [16] study in the leave‐one‐out sensitivity analysis. I 2 indicates heterogeneity across studies. Figure S2: Forest plot showing the pooled effect estimate after excluding Yanovski et al. [15] study in the leave‐one‐out sensitivity analysis. I 2 indicates heterogeneity across studies. Figure S3: Forest plot showing the pooled effect estimate after excluding 12 months outcome from Taş et al. [13] study in the leave‐one‐out sensitivity analysis. I 2 indicates heterogeneity across studies. Figure S4: Forest plot showing the pooled effect estimate after excluding van Der Aa et al. [14] study in the leave‐one‐out sensitivity analysis. I 2 indicates heterogeneity across studies. Figure S5: Forest plot showing the pooled effect estimate after excluding Anderson et al. [12] study in the leave‐one‐out sensitivity analysis. I 2 indicates heterogeneity across studies. [file EDM2-9-e70232-s001.docx]

Table S1: Outcomes of the included studies in this systematic review and meta-analysis

| **Study ID** | | Baseline B12 Levels pg/ml | | B12 Levels Change in 6 months in pg/ml | | | | changes in 12 months in pg/ml | | | | vitamin B12 deficiency after at least 12 months | | | |
| --- | --- | --- | --- | --- | --- | --- | --- | --- | --- | --- | --- | --- | --- | --- | --- |
|  |  | Treatment | | Treatment | | | | Treatment | | | | treatment | | Control | |
|  |  | Mean | SD | Mean | SD | Total | SE | Mean | SD | Total | SE | Event | Total | Event | Total |
| **Anderson et al. ¹²** | | 560.98 | 227.64 | -50.14 | 321.67 | 45 | 47.95 | -90.79 | 383.84 | 45 | 57.22 | 0 | 45 | 0 | 45 |
| **Taş et al. ¹³** | **6months treated group** | 211.38 | 59.62 | -1.35 | 87.24 | 24 | 17.8 |  |  |  |  |  |  |  |  |
|  | **12months treated group** | 222.22 | 50.14 | -14.9 | 66.288 | 11 | 19.99 | -13.55 | 73.835 | 11 | 22.26 | 5 | 12 |  |  |
| **Van der Aa et al. ¹⁴** | | 475.16 | 163.81 |  |  |  |  |  |  |  |  | 3 | 23 | 0 | 19 |
| **Yanovski et al.¹⁵** | | 683 | 270 | -57.1 | 420.46 | 53 | 57.75 |  |  |  |  | 0 | 53 | 0 | 47 |
| **Yu et al. ¹⁶** | |  |  | -31.78 | 293.1 | 151 | 23.85 | -4.92 | 335.71 | 151 | 27.32 | 1 | 151 |  |  |

Abbreviations: SD= standard deviation , SE= standard error


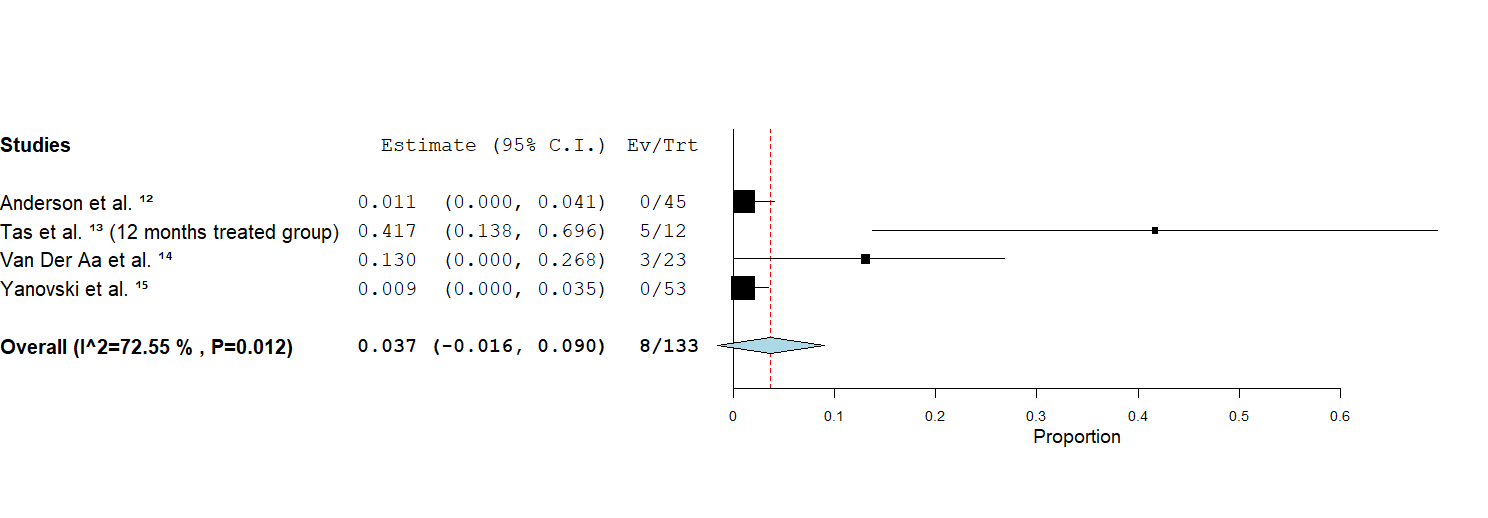
Figure S1. Forest plot showing the pooled effect estimate after excluding Yu et al.**¹⁶** study in the leave-one-out sensitivity analysis. I² indicates heterogeneity across studies.


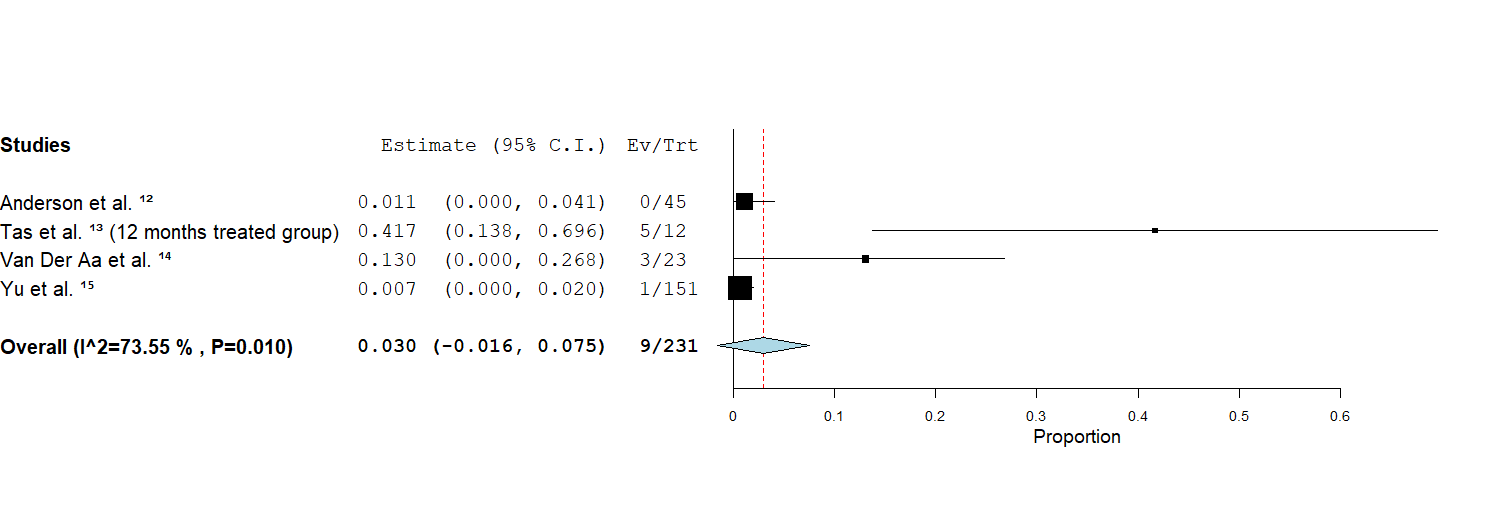
Figure S2. Forest plot showing the pooled effect estimate after excluding Yanovski et al.**¹⁵** study in the leave-one-out sensitivity analysis. I² indicates heterogeneity across studies.


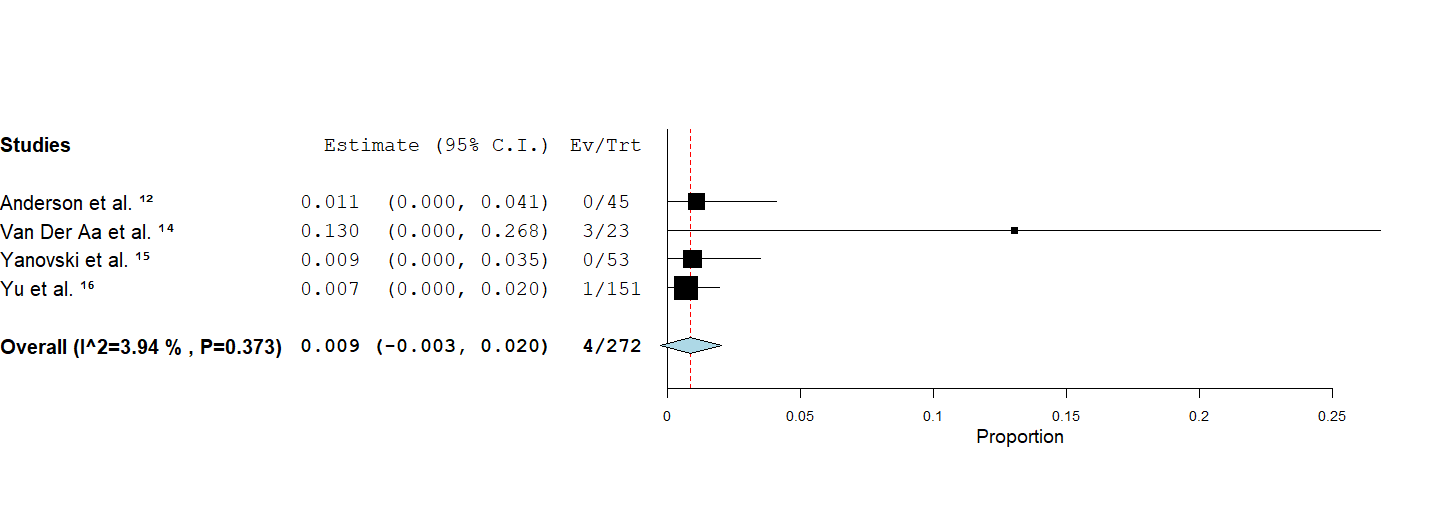


Figure S3. Forest plot showing the pooled effect estimate after excluding 12 months outcome from Tas et al.**¹³** study in the leave-one-out sensitivity analysis. I² indicates heterogeneity across studies.


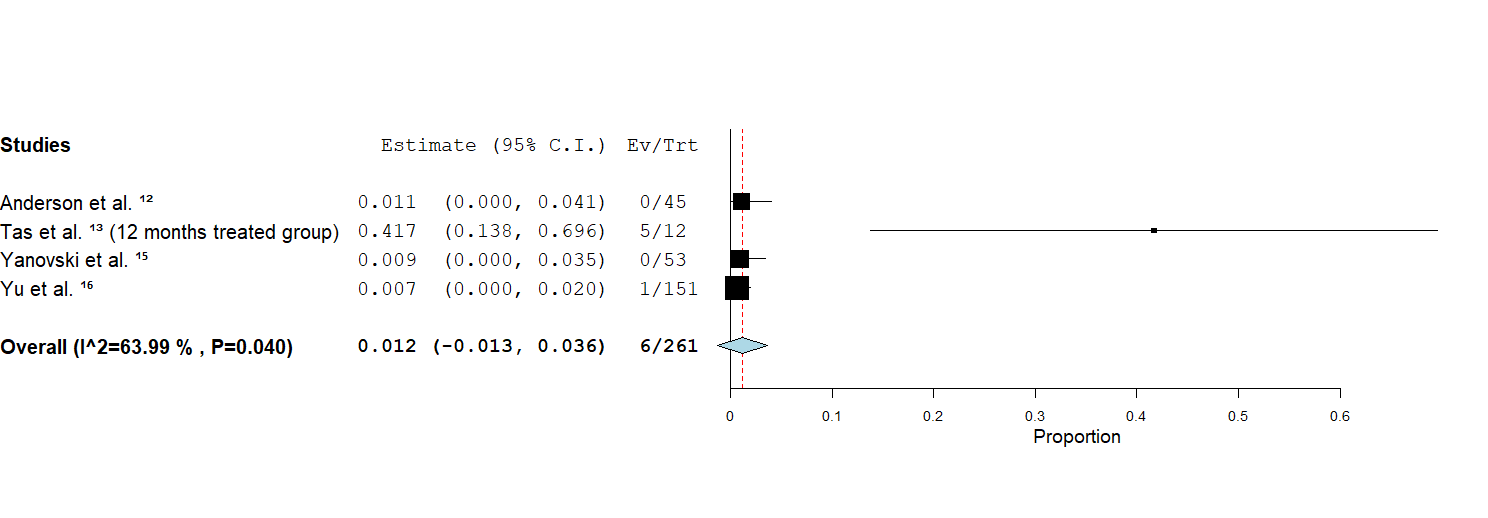


Figure S4. Forest plot showing the pooled effect estimate after excluding Van Der Aa et al.**¹⁴** study in the leave-one-out sensitivity analysis. I² indicates heterogeneity across studies.


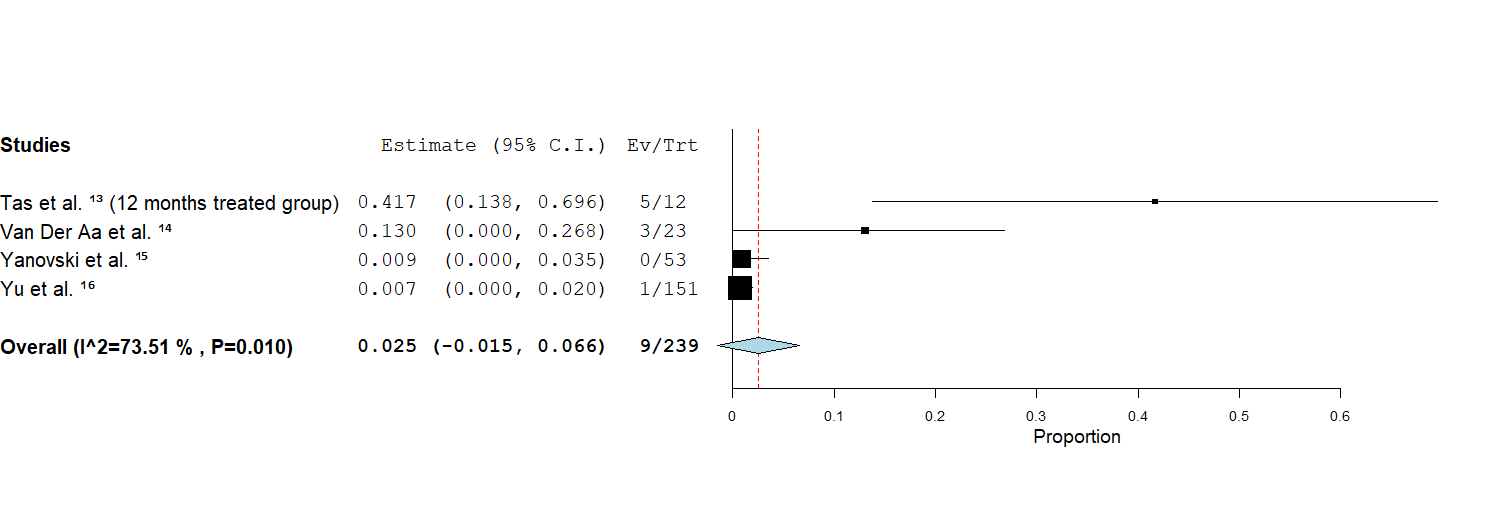
Figure S5. Forest plot showing the pooled effect estimate after excluding Anderson et al.**¹²** study in the leave-one-out sensitivity analysis. I² indicates heterogeneity across studies.
